# Bioactive Compounds Offered in Microcapsules to Determine the Nutritional Value of Copepods’ Natural Diet

**DOI:** 10.3390/md11072459

**Published:** 2013-07-12

**Authors:** Dörthe C. Müller-Navarra, Mark E. Huntley

**Affiliations:** Marine Biology Research Division, Scripps Institution of Oceanography, 0202, La Jolla, CA 92093, USA; E-Mail: meh333@cornell.edu

**Keywords:** bioactive compounds, PUFA, essential fatty acids, microcapsules, zooplankton

## Abstract

Experiments were performed, feeding *Calanus pacificus* seston and a food consisting of seston and microcapsules (μ-caps), *i.e.*, protein and lipid μ-caps to test for potential biochemical limitation. Seston was collected off Scripps Pier (La Jolla, CA, USA). Whereas protein μ-caps were too small to be efficiently ingested, lipid μ-caps rich in ω3-highly-unsaturated fatty acids (ω3-HUFA) were ingested similarly to natural seston and lipids were assimilated. However, egg production experiments exhibited that animals fed with lipid μ-caps didn’t produce significantly more eggs than with seston of equal carbon concentration and egg production even declined when the diet consisted of 50% lipid μ-caps. Thus, the content of certain ω3-HUFA seemed to have been sufficiently high in seston to prevent limitation. Algal counts revealed that seston consisted mainly of plankton rich in those fatty acids, such as cryptophytes, dinoflagellates, diatoms, and ciliates in the edible size range. This might be characteristic for upwelling systems like the area off Southern California which are known for high trophic transfer efficiency.

## 1. Introduction

Bioactive compounds play a central role in shaping marine food webs because they can determine life history traits for all trophic level food web members. They act either as non food biochemical components, such as pheromones or detrimental food components, including polyunsaturated aldehydes (e.g., [1] and references therein) and toxins (e.g., [2,3,4]), or act directly as nutritional bio-chemicals. For the latter, essential food constituents namely essential amino and fatty acids, sterols and vitamins, are important in determining growth and reproduction of heterotrophic consumers and, thus can become critical for trophic transfer within aquatic food webs [5]. Limitations by biochemicals for zooplankton in nature are evidenced indirectly for essential amino acids via homeostatic consideration [6,7], or for ω3-polyunsaturated fatty acids (PUFA) through experiments measuring growth and egg production related to specific fatty acids of the sestonic food [8,9,10,11,12,13,14,15]. Although responses in hatching can also be connected to the presence of certain PUFA, especially DHA [16], the DHA/EPA ratio [13], ALA, EPA and DHA [17], or LIN and ARA [18], there is debate over whether polyunsaturated aldehydes detrimentally effect egg hatching in copepods [1,19,20]. In any case, by applying potential limiting biochemicals major differences in food quality for zooplankton can be explained [18,21,22].

To determine the role of specific biochemical groups contributing to copepod food limitation, we here supplemented seston with micro-capsules (μ-caps). In live food organisms—due to physiological constraints—many biochemical constituents may fluctuate in conjunction, which impedes the ability to test for the effect of single constituents. The use of μ-caps enables defined manipulation of dietary biochemical composition, thus providing a useful tool to assess specific biochemical components as determinants of food quality [23,24] and their role in limiting specific physiological functions. For example, enhanced rates of zooplankton growth or egg production on a μ-cap/seston diet, compared to a natural seston diet of equal carbon concentration, would indicate the specific component offered in the μ-cap was limiting in the seston diet.

In this study we performed egg production experiments, feeding *Calanus pacificus* untreated seston, and seston which was supplemented with two kinds of micro-capsules (μ-caps): (i) lipid μ-caps with a high proportion of ω3-highly-unsaturated fatty acids (especially EPA and DHA) and (ii) protein μ-caps containing bovine serum albumin. We were thus able to alter the biochemical composition of the sestonic diet and to study the effect of ω3-HUFAs or protein alterations on the nutritional value of the natural diet. Egg production experiments are a popular method to study food limitation for zooplankton in general and for marine copepods especially (e.g., [25,26,27,28,29,30,31,32]) and those experiments have already revealed evidence for limitation by certain ω3-PUFA, including ALA and DHA [8,12,13,14].

## 2. Results and Discussion

### 2.1. Uptake and Suitability of μ-Capsules

**Figure 1:**
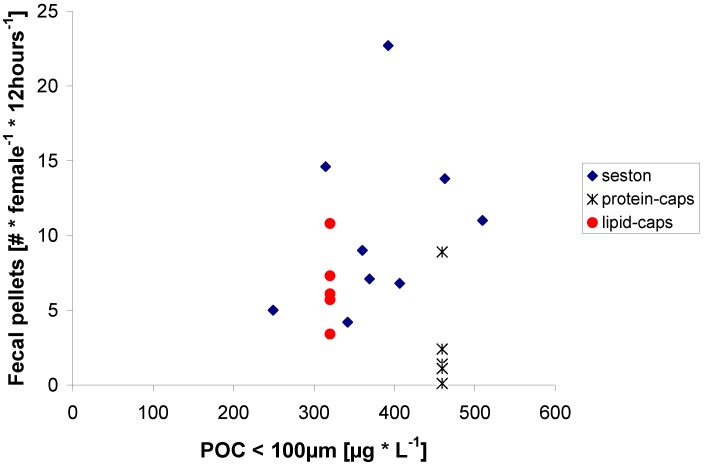
Fecal pellet production by *C. pacificus* as a function of particulate organic carbon content (POC) of the food in three diets.

Preliminary experiments using Nile-red in fecal pellet experiments confirmed that lipid μ-caps were ingested by the copepods. Lipids were apparently removed from the μ-caps during gut passage as no lipid droplets could be seen in the fecal pellets. In some instances, Nile-red stained lipid could even be detected in the eggs. Fecal pellet production was related to the carbon content of the seston food treatments, and fecal pellet production of copepods feeding on lipid μ-caps was in the same range as for seston (Figure 1). In summary, the gelatinous lipid μ-caps were adequately ingested by the copepod and their contents were assimilated, thus demonstrating their suitability for experimental manipulation of the diet.

Our results indicate that gelatinous μ-caps may be a good alternative to liposomes which, though adequate for tracer studies in marine copepods [33], with a mean size of 6 μm [34] are too small to be efficiently ingested by most copepods.

The carbon concentration of the protein μ-caps suspension was well in the range of seston carbon concentrations but fecal pellet production on protein μ-caps was much lower than expected from the carbon content (Figure 1). With a mean diameter of 6.3 μm (Casy Counter), they were probably too small to be efficiently ingested. If we assume an ingestion efficiency of 40% reported for particles of that size [35]—comparable to 180 μg C·L^−1^ in our study—the fecal pellet production would match well.

### 2.2. Egg Production in Relation to Food Concentration

**Figure 2:**
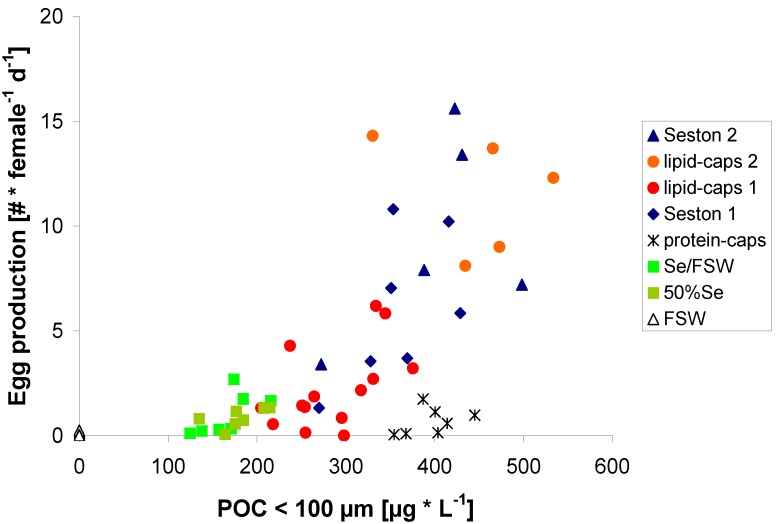
Egg production (E) of *C. pacificus* in relation to the particulate organic carbon content (POC) of the food treatments: [E = 0.019 × POC − 1.8; *p* < 0.001; *r*^2^ = 0.38] [E = 0.024 × POC − 2.4]; *p* < 0.001; *r*^2^ = 0.58, if protein μ-caps treatment is omitted.

Egg production rates of females feeding on seston were in the range found by Mullin [30] for *C. pacificus*, which were fed seston from waters off Point Conception (CA, USA) during July 1989 (2 to 18 eggs female^−1^ day^−1^), using a comparable experimental design. The regression between egg production and food carbon (Figure 2) revealed a threshold concentration for egg production of 100 μg C·L^−1^, below which no eggs were produced.

This threshold value of food concentration for egg production is in the range previously found [36]. Egg production in our study was always below the critical concentration for maximal egg production, emphasizing food limiting conditions for *C. pacificus* during this time of the year, which seems to be common for planktonic calanoid copepods [13,18,25,26,27,29,32]. However, the critical concentration must have been above 400 μg C L^−1^, which is high compared to levels measured with cultured algae [37], but in the range of what has been measured with seston as food [15,38]. This may indicate that the seston, in contrast with many cultured food algae, consisted also of a significant share of algae which were too small (Table 1) to be efficiently ingested by *C. pacificus* [35,39]. Thus, accessible carbon to *C. pacificus* was considerably lower than the total measured carbon concentration.

**Table 1:** Algal composition of seston during the experiments. * Most diatom frustules were unfortunately partly dissolved prior to counting.

Size	Respresentative organisms	Cell volume	Cell volume
[μm]		[μm^3^·mL^−1^]	%
<4	Flagellates	56,651	56.8
4–8	cryptophyte, thecate dinoflagellates	3213	3.2
8–18	*Scrippsiella*, *Prorocentrum* Diatoms *, ciliates	5819	5.8
>18	*Protoperidinium*, *Prorocentrum*, *Nitzschia*	34,106	34.2

**Figure 3:**
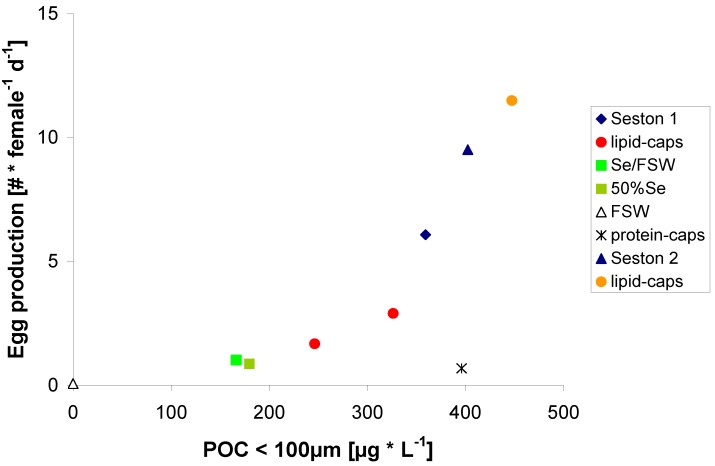
Mean egg production (E) of *C. pacificus* related to the mean organic carbon content (POC) of the food treatments: [E = 0.022 × POC − 2.1]; *p* < 0.05; *r*^2^ = 0.49; [E = 0.024 × POC − 2.4]; *p* < 0.01; *r*^2^ = 0.73, if protein μ-caps treatment is omitted.

Egg production increased with increasing carbon concentration, but a high degree of scatter was obtained although each data point represents the mean value for observations from nearly 50 females (Figure 2). Calculating the mean values for each treatment (except the protein μ-cap treatment) over the whole experimental duration diminished a large amount of scatter, *i.e.*, explained variability increased from *r*^2^ = 0.57 to *r*^2^ = 0.76 using a linear regression (Figure 3).

Besides diminishing differences in prehistory and aging of the females by this procedure, variable time lags between food ingestion and egg laying were probably the main source of variability when considering daily egg production rates. Averaging over 24 h may be too short to include the full cycle, especially at low egg production rates where egg laying frequency can be longer than one day.

**Figure 4:**
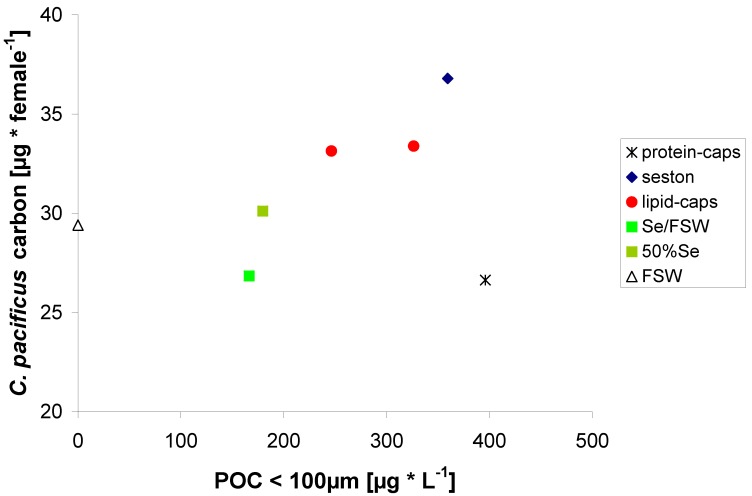
Relationship between mean food carbon of the different treatments and the carbon content of *C.* *pacificus* at the end of the experiment.

The exponential shape of the relationship [E = 0.17 × e^(0.0096 × POC)^]—again excluding the protein μ-caps treatment—may be due to weight differences in *C. pacificus* (*i.e.*, carbon content) which developed over the experimental duration depending on the carbon content of their food (Figure 4).

### 2.3. Egg Production on Protein μ-Caps

Egg production rates on a diet of protein capsules and seston were statistically different from the other treatments (*p* < 0.0001, ANCOVA, Table 1) and *ca.* 40% lower than expected from the food carbon concentration, which agrees with a lower ingestion efficiency [35], also indicated by lower fecal pellet production. Thus, lower ingestion rates were probably the reason for the observed lower egg production rates in relation to the food carbon.

### 2.4. Egg Production on Lipid μ-Caps Rich in ω3-HUFA

Females fed a combination of lipid μ-caps and seston had similar egg production rates per food carbon as those fed natural seston only. Substitution of seston with lipid μ-caps of high EPA and DHA content, amounting to 25% ω3-HUFA per lipid μ-cap carbon (Table 2), neither enhanced nor reduced egg production rates over what would have been expected from the food carbon content. Thus, despite comparable ingestion, amendment of the diet with ω3-HUFAs did not enhance sestonic food quality in this study. Rather, it was lipid carbon, not the presence of essential ω3-HUFA, that explained differences in *C. pacificus* egg production. Thus, accessible food carbon rather than ω3-HUFA appears to have limited *C. pacificus* female egg production. Microscopic examination revealed that the surface seston assemblage consisted of cryptophytes, dinoflagellates, diatoms and ciliates (Table 1) which were probably rich in ω3-HUFAs and biochemically balanced [40].

**Table 2:** Fatty acid composition of the lipid μ-caps as percent of total fatty acids; n.d., not detected. ? = position unknown.

Fatty acid	%
C14:0	5.83
C14:1ω?	0.21
C14:1ω5	0.13
C15:0	0.47
C16:0	13.98
C16:1ω7	6.83
C16:1ω5	0.51
C16:2ω6	0.07
C16:3ω4	1.24
C16:4ω1	0.82
C17:0	0.6
C18:0	2.83
C18:1ω12/ω9	14.86
C18:1ω7	3.59
C18:2ω6	2.95
C18:3ω6	0.26
C18:3ω3	1.74
C18:4ω3	5.59
C20:0	0.32
C20:1ω9	n.d.
C20:1ω7	0.45
C20:2ω6	0.17
C20:3ω6	0.17
C20:4ω6	0.91
C20:5ω3	13.36
C22:0	n.d.
C22:1ω9	10.71
C22:6ω3	10.05
C23:0	0.21
C24:0	0.05
C24:1ω9	0.79
∑HUFA	24.7

**Figure 5:**
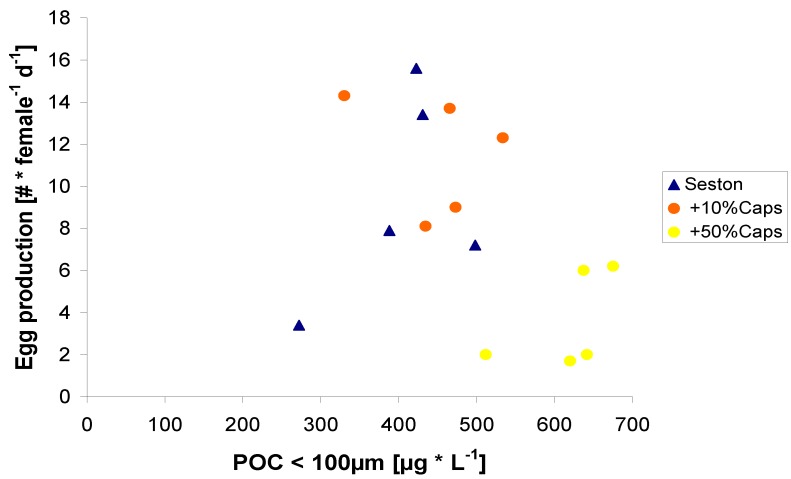
Egg production of *C. pacificus* related to the particulate carbon content (POC) of the food from experiment 2.

Further lipid additions unbalanced the food composition and negatively affected egg production rates. An addition of 50% lipid μ-caps caused egg production to decrease to less than half of what was measured on the seston diet alone (Figure 5). Thus, the lipid μ-caps at this concentration induced a negative effect on egg production. This indicates that a dietary lipid content of over 50% or an ω3-HUFA share of over 25% must have unbalanced the biochemical composition to a degree that was not only suboptimal but even detrimental for *C. pacificus*’ egg production. Especially ω3-HUFA with 5 or 6 double bonds, such as EPA, may have toxic effects at high concentrations in algae [41]. We did not analyze the fatty acid content of the seston used in our experiments, however seston in comparable studies contain between 1.7% and 11% HUFA (as percentage of total fatty acids), and lipid content generally lies in the range between 2.5% and 12.3% of total seston carbon [42,43,44,45]. Thus ω3-HUFA per carbon was probably <1% in the seston compared to 25% in the lipid capsules. Addition of 50% lipid μ-caps thus increased the ω3-HUFA content *ca.* 10-fold, from less than 1% to over 10%., which is even higher than found for most ω3-HUFA rich diatom cultures [46,47].

### 2.5. Zooplankton Nutrition and the Coupling between Primary and Secondary Production

Limitation of zooplankton egg production by PUFA has been observed in bays [8,12], fjords [14,20], shelf seas [13], and in lakes [9,10,11]. By contrast, our study suggests that in up-welling systems such limitation of egg production may play a minor role, since the seston is generally rich in ω3-HUFA, especially EPA and DHA. Due to the sustained input of new nutrients into the euphotic zone that is characteristic of upwelling areas, nutrient saturated conditions of algae prevail, yielding algae that are nutritionally superior to those which are nutrient limited [46,47,48]. In addition, upwelling conditions favor the occurrence of diatoms [49,50], which are specifically rich in ω3-HUFA such as EPA [46,47]. Thus, under upwelling conditions, levels of ω3-HUFA in the seston probably exceed those required by copepods, leading to limitation by protozoan and algal biomass [25] and permitting efficient carbon transfer between primary and secondary production [51] which further sustains high fish production [52,53]. On the contrary, under stratified conditions, due to lack of mixing and silicate inputs, food quality is comparatively low [28,31] and carbon or energy transfer efficiency will therefore also be low. Seston in those systems consists of detritus and non-diatom phytoplankton species which are nutrient limited and slow growing, leading to prevalent biochemical (e.g., by certain ω3-HUFA) limitation. Apparently different nutrient regimes favor different types of zooplankton food limitation which also impact trophic transfer in aquatic systems.

## 3. Experimental Section

### 3.1. Animal Collection and Experimental Protocol

*Calanus pacificus* were caught using vertical net tows (500 μm) in the water off Scripps pier (La Jolla, CA, USA). Females were sorted in the laboratory and collected in 2 L jars (for roughly 6 h) before being transferred to 500 mL egg-production chambers. Then the animals were split into groups and acclimatized for an additional 16 h by pre-feeding them their respective experimental diets. Acclimation and the experiments were performed in a constant temperature walk-in chamber at 18 °C. Although eggs were counted during that first day, the results were omitted from the analyses, since those eggs might have resulted from food ingested prior to the experiment [54,55]. The 500 mL incubation chambers were equipped with 100 μm mesh bottom inserts, so that eggs could sink through and be protected from cannibalism. Two experiments were carried out in which the copepods were subjected to various food treatments, which comprised natural seston, diluted seston, filtered sea water, and combinations of seston and either protein-(bovine-serum-albumin) or lipid-(fish-liver-oil) micro-capsules. Experiments were run for 7 days (experiment 1) and 5 days (experiment 2) in the dark. The seston food was prepared from surface water which was collected twice daily from Scripps pier and screened through a 100 μm mesh to remove larger zooplankton. Each time the water was exchanged, eggs and fecal pellets from each chamber were collected on a 50 μm screen and counted.

### 3.2. Micro-Capsule Preparation and Their Ingestion

Lipid μ-caps were prepared with fish-liver-oil before the experiments as described by Cary *et al*. [56]. Protein μ-caps were prepared with bovine-serum-albumin according to Langdon & DeBevoise [57]. By offering *Calanus pacificus* lipid μ-caps stained with Nile-red we could test for the ingestion and assimilation of the lipid μ-caps. In addition, fecal pellet production was used as an indirect measure of ingestion. Fecal pellet production was determined during the 1st experiment by collecting and counting fecal pellets at each water exchange, *i.e.*, every 12 h. As μ-caps were offered during night and seston during day integrated 12 h values were obtained which were related to the food carbon of the food offered (seston, lipid μ-caps and protein μ-caps, respectively).

### 3.3. Egg Production Measurements

Two experiments were performed between June 30, and July 6 (experiment 1), and between 12 and 17 September 1995 (experiment 2), respectively. Experiment 1 included 7 treatments, with *circa* 16 replicates each. Each egg-production chamber (replicate) contained 3 *C. pacificus* females. Water was exchanged every 12 h. The following treatments were employed: (1) seston (24 h); (2) 50% seston (diluted with filtered sea water, 24 h); (3) filtered sea water (FSW, 24 h); (4) FSW (night—12 h)/seston (day—12 h); (5) 160 μg C L^−1^ lipid-caps (night—12 h)/seston (day—12 h); (6) 320 μg C L^−1^ lipid-caps (night—12 h)/seston (day—12 h) and (7) protein-caps (night—12 h)/seston (day—12 h). Since diel rhythms are a common phenomenon, we averaged food carbon concentrations and egg production values over 24 h periods, before relating the two parameters using regression analysis. At the end of experiment 1, experimental females were also analyzed for CHN. In the second experiment, only 1 female was placed in each egg-production chamber. 12 replicates were used per treatment. The following treatments were employed: (1) unaltered seston for 24 h, (2) seston + lipid μ-caps at a concentration of 10% seston carbon for 24 h, and (3) lipid μ-caps at a concentration of 50% seston carbon for 24 h. The water was exchanged every 24 h.

### 3.4. Seston Analyses

At each water exchange, an aliquot of the water which was screened through a 100 μm net and used for the experiment, was filtered through precombusted GF/C filters. The filters were dried at 60 °C overnight and stored in a dessicator until C/N analyses (Carlo-Erba Analyser) were performed. At various times during the experiments, water was removed for preservation with Lugol’s solution. 100 mL of sample was settled and examined under an inverted microscope.

## 4. Conclusions

In contrast to the protein μ-caps, lipid μ-caps were well suited to be used in nutrition experiments with copepods. Negative effects were observed when 50% of the food carbon consisted of lipid μ-caps, which are a sign of biochemical imbalance and should be considered in dietary studies. The lack of a clear beneficial dietary effect of ω3-HUFA (EPA, DHA) supplementation at lower concentrations needs further studies but agrees well with the high food quality of taxa representing the seston of edible size range, such as flagellates (cryptophytes, dinophytes), diatoms, and ciliates. This seston composition is specific to the oceanographic situation off La Jolla, California, an upwelling area. Our results suggest that the high ω3-HUFA content of seston may contribute significantly to the high trophic transfer efficiency that is characteristic of upwelling areas.

## Abbreviations

DHA: docosahexaenoic acid
DW: dry weight
E: egg production
EPA: eicosapentaenoic acid
FA: fatty acid
FAME: fatty acid methyl ester
FSW: filtered sea water
GC-FID: gas chromatography-flame-ionisation detector
HUFA: highly unsaturated fatty acid
μ-caps: microcapsules
POC: particulate organic carbon
PUFA: polyunsaturated fatty acid
